# Efficient affinity maturation of antibody variable domains requires co-selection of compensatory mutations to maintain thermodynamic stability

**DOI:** 10.1038/srep45259

**Published:** 2017-03-28

**Authors:** Mark C. Julian, Lijuan Li, Shekhar Garde, Rebecca Wilen, Peter M. Tessier

**Affiliations:** 1 Center for Biotechnology & Interdisciplinary Studies, Isermann Dept. of Chemical & Biological Engineering, Rensselaer Polytechnic Institute, Troy, NY 12180, USA.

## Abstract

The ability of antibodies to accumulate affinity-enhancing mutations in their complementarity-determining regions (CDRs) without compromising thermodynamic stability is critical to their natural function. However, it is unclear if affinity mutations in the hypervariable CDRs generally impact antibody stability and to what extent additional compensatory mutations are required to maintain stability during affinity maturation. Here we have experimentally and computationally evaluated the functional contributions of mutations acquired by a human variable (V_H_) domain that was evolved using strong selections for enhanced stability and affinity for the Alzheimer’s Aβ42 peptide. Interestingly, half of the key affinity mutations in the CDRs were destabilizing. Moreover, the destabilizing effects of these mutations were compensated for by a subset of the affinity mutations that were also stabilizing. Our findings demonstrate that the accumulation of both affinity and stability mutations is necessary to maintain thermodynamic stability during extensive mutagenesis and affinity maturation *in vitro*, which is similar to findings for natural antibodies that are subjected to somatic hypermutation *in vivo*. These findings for diverse antibodies and antibody fragments specific for unrelated antigens suggest that the formation of the antigen-binding site is generally a destabilizing process and that co-enrichment for compensatory mutations is critical for maintaining thermodynamic stability.

The intimate connection between protein structure and function means that attempts to alter one typically require changes in the other. Many previous studies have demonstrated that modifying protein sequences to improve their existing functions or introduce new ones often leads to structural changes that are destabilizing^1^,^2^,^3^. This has been elegantly demonstrated in directed evolution studies of enzymes^4^,^5^,^6^,^7^. Enhancement or alteration of enzyme function is typically accompanied by a reduction in stability, and it is often necessary to introduce stabilizing mutations to compensate for such activity/stability trade-offs^5^,^8^,^9^,^10^,^11^.

To what extent the activity/stability trade-offs observed for enzymes also occur for antibodies is less well understood. The variable regions of antibodies are subjected to significant mutagenesis during somatic hypermutation, which leads to the accumulation of mutations in the complementarity-determining regions (CDRs) as well as in the framework regions^12^,^13^,^14^. Conventional wisdom suggests that mutations in the hypervariable CDRs weakly impact antibody stability. However, the fact that the six CDRs are highly structured and packed together in close proximity to form the antigen-binding site suggests that antibodies may not be able to accumulate multiple affinity-enhancing mutations in their CDRs without being destabilized. Indeed, recent studies suggest that natural antibodies acquire stability mutations during somatic hypermutation to maintain thermodynamic stability^15^,^16^.

We have recently observed strong affinity/stability trade-offs during the directed evolution of a human antibody (V_H_) domain specific for the Alzheimer’s Aβ42 peptide^17^. We generated mutant libraries of a stable V_H_ antibody scaffold (B1a^18^) that was initially grafted with Aβ residues 33–42 in CDR3, displayed the mutants on the surface of yeast, and selected for V_H_ domains with improved binding affinity. Interestingly, V_H_ domains with improved affinity were typically destabilized. Accumulation of several affinity mutations led to V_H_ domains that were destabilized on the surface of yeast and unfolded as autonomous domains when produced in bacteria.

To overcome these strong affinity/stability trade-offs, we adapted conventional yeast surface display^19^,^20^,^21^ to enable co-selection of high antibody stability in addition to high affinity. We replaced the conventional display antibody that recognizes a linear epitope tag at the C-terminus of unfolded and folded V_H_ domains on the surface of yeast with a conformational ligand (Protein A) that is specific for folded V_H_ domains. This approach led to the isolation of a stable Aβ-specific V_H_ domain (referred to as P4) with several mutations in the CDRs and framework regions (apparent melting temperature of 66 °C). The fact that the V_H_ domain retained high stability despite being heavily mutated (12 mutations, ~9% of the V_H_ domain) – which was not possible in the absence of strong co-selections for enhanced stability – led us to posit that our directed evolution approach may be mimicking the natural process of somatic hypermutation by accumulating both affinity and stability mutations^15^,^16^. These results also suggest that the formation of the antigen-binding site in human variable domains may generally be a destabilizing process. Here we test these hypotheses via extensive mutagenesis and functional analysis of an evolved human variable domain using both experimental and computational methods.

## Results

### Affinity-enhancing mutations can increase or decrease thermodynamic stability of antibody variable domains

**Figure 1:**
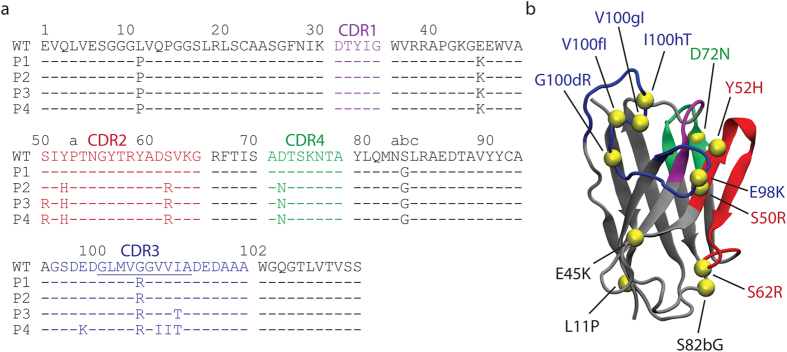
Sequences of evolved human V_H_ domains and structural model of the most evolved variant. (**a**) Amino acid sequences of the wild-type V_H_ domain with Aβ residues 33–42 (underlined) grafted into CDR3 and evolved variants (P1-P4) selected for enhanced affinity and stability. The CDR regions (as defined by Kabat) are highlighted in purple (CDR1), red (CDR2), blue (CDR3) and green (CDR4). (**b**) Modeled structure of the evolved P4 variant using the crystal structure of the parental V_H_ domain (B1a, PDB: 3B9V) and molecular dynamics simulations. The V_H_ domains also contain three FLAG-tags and a heptahistidine tag at the C-terminus as well as additional N-terminal residues (Met-Ser-Lys-Leu for WT and Met-Ser-Ala-Ser for P1-P4), which are omitted for clarity.

The antibody (V_H_) domains that we isolated by co-selecting for affinity and stability^17^ are summarized in Fig. 1. These evolved V_H_ domains contain 4 (P1), 7 (P2), 9 (P3) and 12 (P4) mutations in the framework and CDR regions that were accumulated sequentially during successive rounds of mutation and selection. One V_H_ loop (residues 71–78) near the conventional CDRs (referred to as CDR4)^22^ was also mutated. The mutations are primarily located on the former V_H_/V_L_ interface (Fig. 1b).

**Figure 2:**
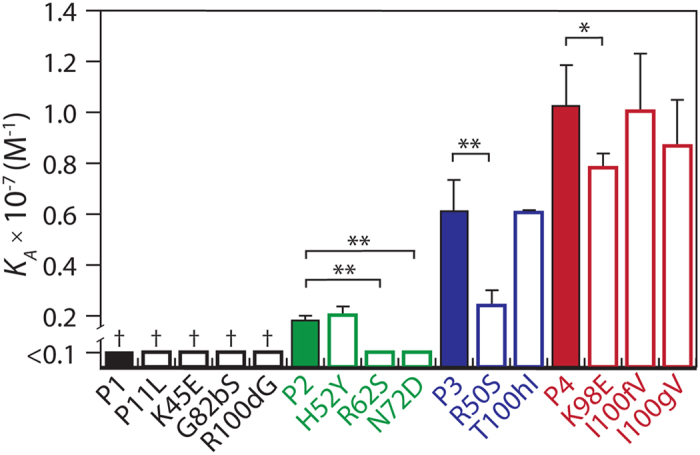
Analysis of binding affinity for P-series V_H_ variants with single wild-type reversion mutations. The equilibrium association constant (*K*_*A*_) values of V_H_ variants containing single reversion mutations were evaluated using yeast surface display and flow cytometry (PBS + 1 g/L BSA). The reversion mutations are highlighted in black (P1), green (P2), blue (P3) and red (P4). The measurements are averages of multiple independent experiments (n = 3–7) and the error bars are standard deviations. A two-tailed Student’s *t*-test was used to judge statistical significance [*p*-values < 0.05 (*) or 0.01 (**)]. The statistical significance of P1 and its associated reversion mutants (P11L, K45E, G82bS and R100dG; †) could not be computed because of the low affinity of these variants.

To evaluate potential affinity/stability trade-offs for these V_H_ domains, we first evaluated the affinity of each variant (P1, P2, P3 and P4) as well as single reversion mutants of each variant (e.g., four P1 variants each with a single reversion mutation to the corresponding wild-type residue; Fig. 2). This allowed us to examine not only increases in V_H_ affinity along the evolutionary pathway for the P-series V_H_ domains, but also the contribution of each mutation to the overall affinity in the context in which it was acquired. The equilibrium association constant (*K*_A_) values for P1 and its single reversion mutants were not measurable due to their low affinity. However, the P2 V_H_ domain displayed enhanced affinity relative to P1. Two of the P2 mutations (R62 and N72) were important for affinity (*p*-values < 0.0004), as their reversion mutations (S62 and D72) reduced binding below detectable levels. In contrast, the other P2 mutation (H52) weakly impacted affinity (*p*-value of 0.31). The affinity of P3 was significantly increased relative to P2 (*p*-value of 0.0006). One of the P3 mutations (R50) also contributed significantly to affinity (*p*-value of 0.0005), while the other (T100h) did not (*p*-value of 0.95). Finally, the P4 affinity was further enhanced relative to P3 (*p*-value of 0.0003). One of the P4 mutations (K98) contributed to increased affinity (*p*-value of 0.03), while the other two P4 mutations (I100f, I100g) did not (*p*-values > 0.1).

**Figure 3:**
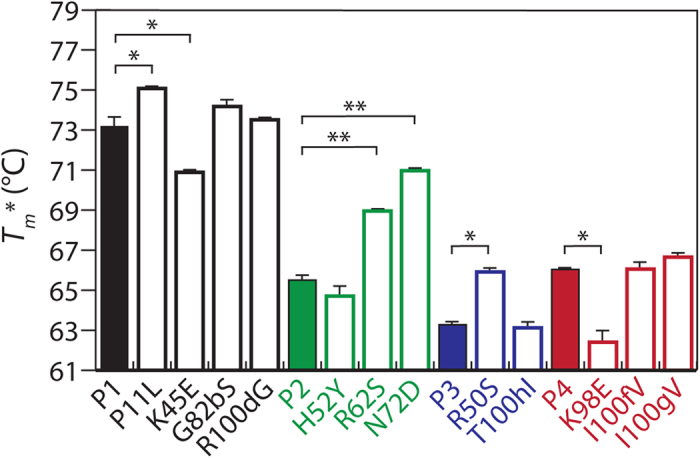
Thermodynamic stability analysis of P-series V_H_ variants with single wild-type reversion mutations. The stability of V_H_ domains containing single reversion mutations were evaluated using autonomous V_H_ domains produced in bacteria. Apparent melting temperature (*T*_m_^*^) values were calculated by monitoring the circular dichroism signal at 235 nm during thermal unfolding. The reversion mutations are highlighted in black (P1), green (P2), blue (P3) and red (P4). The measurements are averages of two repeats and the error bars are standard deviations. A two-tailed Student’s *t*-test was used to judge statistical significance [*p*-values < 0.05 (*) or 0.01 (**)].

These findings – which reveal a subset of accumulated mutations that contribute disproportionally to affinity – led us to investigate if the same or different mutations impact antibody stability. Therefore, we produced the V_H_ mutants as autonomous domains in bacteria, and obtained high purification yields (10–80 mg/L of culture) and purities (Fig. S1). Stability analysis of the V_H_ domains revealed unique roles of different V_H_ mutations acquired during affinity maturation (Figs 3 and S2). The stability of the P1 variant was similar to wild-type (apparent *T*_*m*_ of 73.2 ± 0.5 °C for P1 relative to 75.0 ± 0.3 °C for wild-type; *p*-value of 0.06). Interestingly, the P1 mutation K45 – a framework mutation located at the base of the β-strand leading to CDR2 – was stabilizing, as the reversion mutation E45 significantly reduced stability (apparent *T*_*m*_ of 70.9 ± 0.1 °C relative to 75.0 ± 0.3 °C for wild-type; *p*-value of 0.02). In contrast, the P1 mutation P11 was destabilizing (*p*-value of 0.04), and the other P1 mutations (R100d and G82b) did not significantly impact stability (*p*-values > 0.15).

The variable impact of the P1 mutations on stability was also seen for the other P-series V_H_ variants (Fig. 3). The stability of the P2 variant was reduced relative to P1 (apparent *T*_*m*_ of 65.5 ± 0.2 °C for P2 relative to 73.2 ± 0.5 °C for P1; *p*-value of 0.003). This was primarily due to two destabilizing P2 mutations (R62 and N72), as their reversion mutations (S62 and D72) were stabilizing (*p*-values < 0.003). Likewise, the stability of P3 was further reduced relative to P2 (apparent *T*_*m*_ of 63.3 ± 0.1 °C for P3 relative to 65.5 ± 0.2 °C for P2; *p*-value of 0.008), and this was primarily due to the destabilizing mutation R50 (*p*-value of 0.004). Interestingly, the stability of P4 was increased relative to P3 (apparent *T*_*m*_ of 66.0 ± 0.1 °C for P4 relative to 63.3 ± 0.1 °C for P3; *p*-value of 0.002), and this was due to the stabilizing mutation K98 located at the N-terminus of CDR3 (apparent *T*_*m*_ of 62.4 ± 0.6 °C for E98 relative to 66.0 ± 0.1 °C for K98; *p*-value of 0.01). Collectively, these results demonstrate that V_H_ mutations acquired during affinity maturation – especially those that are most important for affinity – can contribute both positively and negatively to thermodynamic stability.

### Evolved antibody variable domains display affinity/stability trade-offs

**Figure 4:**
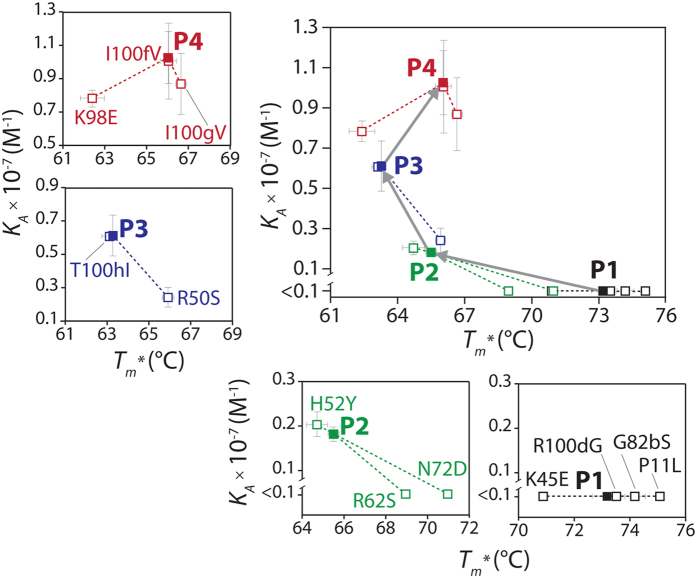
Comparison of the impact of single wild-type reversion mutations on the binding affinity and thermodynamic stability of the P-series V_H_ variants. The affinity and stability measurements for the P-series variants containing single wild-type reversion mutations were evaluated as described in Figs 2 and 3, respectively. Decreases in V_H_ affinity or stability due to reversion mutations signify that the P-series mutations contribute positively to affinity or stability in the corresponding V_H_ domain (and vice versa for increases in V_H_ affinity or stability due to reversion mutations). The reversion mutations are highlighted in black (P1), green (P2), blue (P3) and red (P4). Error bars are standard deviations for two (stability) or three to seven (affinity) independent experiments.

A direct comparison of the impacts of each mutation on the affinity and stability of the V_H_ domains during affinity maturation reveals interesting affinity/stability trade-offs (Fig. 4). It is notable that the gain in affinity for the P2 variant was due to two destabilizing mutations (R62 and N72). This same pattern was seen for the P3 variant, as the gain in affinity was due to a destabilizing mutation (R50). However, a mutation in P4 (K98) contributed positively both to affinity and stability, and resulted in increased stability of P4 relative to P3. These findings demonstrate that affinity-enhancing mutations can reduce the stability of evolved V_H_ domains, and that these destabilizing effects are offset by compensatory mutations in the P-series domains.

**Figure 5:**
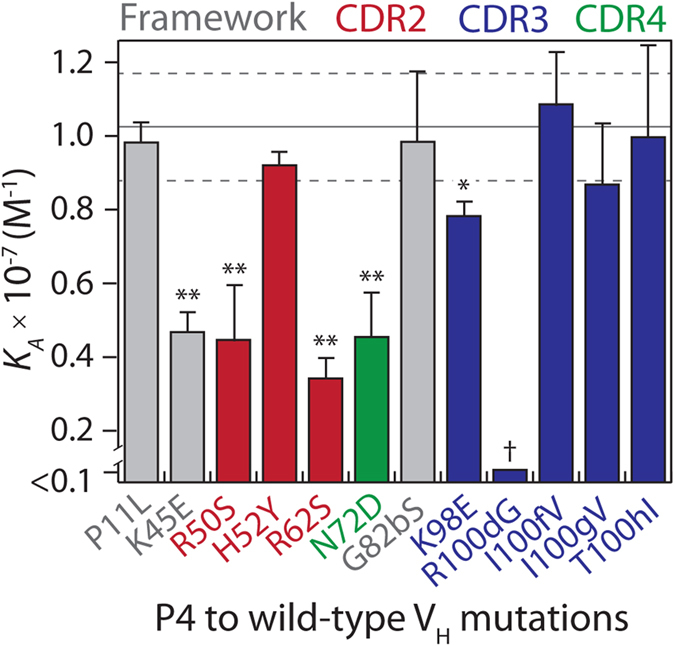
Analysis of binding affinity for P4 V_H_ variants with single wild-type reversion mutations. The equilibrium association constant (*K*_*A*_) values of P4 V_H_ variants containing single wild-type reversion mutations were evaluated as described in Fig. 2. The reversion mutations are highlighted in grey (framework residue), red (CDR2), blue (CDR3) and green (CDR4). The affinity measurements are averages of multiple independent experiments (n = 3–5) and the error bars are standard deviations. The solid and dotted lines represent the average and standard deviation of the P4 V_H_ measurements (n = 7), respectively. A two-tailed Student’s *t*-test was used to judge statistical significance [*p*-values < 0.05 (*) or 0.01 (**)]. The statistical significance of the R100dG reversion mutation (†) could not be computed because of the low affinity of this variant.

We next sought to understand in greater detail the role of mutations acquired at different stages of affinity maturation on affinity/stability trade-offs for the most evolved V_H_ domain with twelve mutations (P4). Therefore, we measured the equilibrium association constants for the P4 variants containing twelve single reversion mutations (Fig. 5). Notably, six mutations contributed significantly to P4 binding (K98 as stated above; K45 in the scaffold, R50 and R62 in CDR2, N72 in CDR4, and R100d in CDR3, *p*-values < 0.0003). Interestingly, the most important affinity mutation (R100d) occurred in P1 and the reversion mutation G100d in P4 reduced binding below detectable levels.

**Figure 6:**
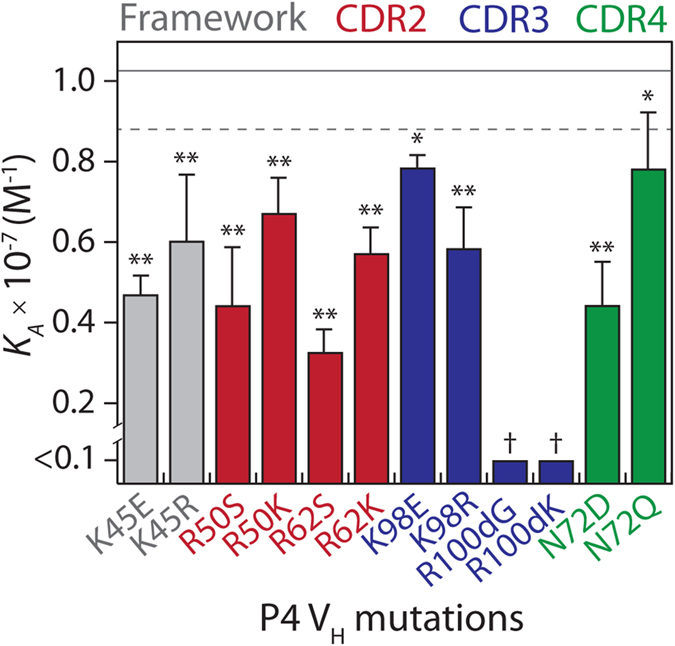
Sensitivity of affinity mutations in the P4 V_H_ domain to arginine-to-lysine, lysine-to-arginine or asparagine-to-glutamine substitution mutations. The three arginine mutations in P4 (R50, R62 and R100d) were reverted individually to the wild-type residue (S50, S62 and G100d) or lysine (K50, K62 and K100d). Two lysine mutations (K45 and K98) were reverted individually to the wild-type residue (E45 and E98) or arginine (R45 and R98). Additionally, the asparagine mutation (N72) is located in an N-linked glycosylation site and was mutated to either aspartic acid (wild-type) or glutamine. The association constant (*K*_*A*_) values of the mutants were evaluated as described in Fig. 2. The mutants are highlighted in grey (framework residue), red (CDR2), blue (CDR3) and green (CDR4). The solid and dotted lines represent the average and standard deviation of the P4 V_H_ measurements (n = 7). The measurements for the P4 mutants are averages of multiple independent experiments (n = 4–5), and the error bars are the standard deviations. A two-tailed Student’s *t*-test was used to judge statistical significance [*p*-values < 0.05 (*) or 0.01 (**)]. The statistical significance of the R100dG and R100dK reversion mutations (†) could not be computed because of the low affinity of these variants.

Three of the key P4 affinity mutations involve introduction of arginine (R50, R62 and R100d), which suggested that electrostatic and/or non-electrostatic interactions involving arginine side chains may be important for antigen binding. To examine the role of arginine mutations in P4 binding, we created three additional mutants – each with a lysine mutation in place of the arginine mutation acquired in P4 – and measured their affinity (Fig. 6). Interestingly, the lysine variants failed to restore binding to P4 levels (*p*-values < 0.002), and the K100d mutation failed to restore binding even to detectable levels.

Two of the other P4 affinity mutations were charge reversal mutations (E45K and E98K), and we tested whether arginine could substitute for lysine at these positions without reducing affinity (Fig. 6). Interestingly, both R45 and R98 reduced affinity relative to wild-type P4 with K45 and K98 (*p*-values < 0.004). These findings reveal that lysine mutations are superior for affinity at some positions in the evolved V_H_ domain relative to arginine mutations (K45 and K98), while arginine mutations are superior at other positions (R50, R62 and R100d).

Another important affinity mutation in P4 is N72, which is located in CDR4 (Fig. 1). Interestingly, mutating aspartic acid to asparagine introduces an N-linked glycosylation site in P4. Given that our directed evolution platform involves a eukaryotic expression host (*S. cerevisiae*), we tested whether the evolved P4 variant was glycosylated. Indeed, after expressing both the P4 and wild-type domains as autonomous V_H_ antibodies in *S. cerevisiae*, we found that the P4 domain was only detectable via SDS-PAGE after treatment with a deglycosylation enzyme specific for *N*-linked glycans (PNGase F; Fig. S3). In contrast, treatment with an enzyme specific for *O*-linked glycans (*O*-Glycosidase) failed to lead to detectable P4 V_H_ domain, while the size and detection sensitivity of the wild-type V_H_ domain was unchanged in the absence or presence of either enzyme.

We also sought to determine if the glycans on P4 were an important factor in mediating antigen binding. To test this, we generated a mutant version of P4 in which the asparagine at position 72 was mutated to glutamine (Q72) to eliminate its N-linked glycosylation site. We found that the Q72 mutant of P4 showed modestly reduced affinity relative to P4 (N72; *p*-value of 0.02) and higher affinity than the D72 mutant (*p*-value of 0.006; Fig. 6). Collectively, these results suggest that P4 is glycosylated on the surface of yeast and that the glycans play a relatively minor role in antigen binding.

Finally, we tested whether the six affinity mutations in P4 (K45, R50, R62, N72, K98 and R100d) could together confer similar affinity as the twelve mutations in P4. Interestingly, the wild-type scaffold with the six affinity mutations displayed significantly lower affinity than P4 (>4 fold reduction in *K*_*A*_, *p*-value of 10^−6^; data not shown). This finding highlights the importance of higher order interactions between mutations acquired during affinity maturation that are not readily identified using single reversion mutations.

### Computational analysis of wild-type and evolved V_H_ domains

To gain further insight into how the P4 mutations alter the hydrophobic and electrostatic properties of the wild-type variable domain, we used computational methods to model both the wild-type and P4 variable domains. The structure of each domain was modeled by adding the appropriate mutations to the parent V_H_ domain structure (B1a, PDB: 3B9V^18^), and performing molecular dynamics simulations and analysis using clustering techniques. The backbone conformations of the V_H_ domains changed modestly during the simulations (100 ns; Fig. S4), as more than 90% of the structures fell within the same cluster (data not shown).

**Figure 7:**
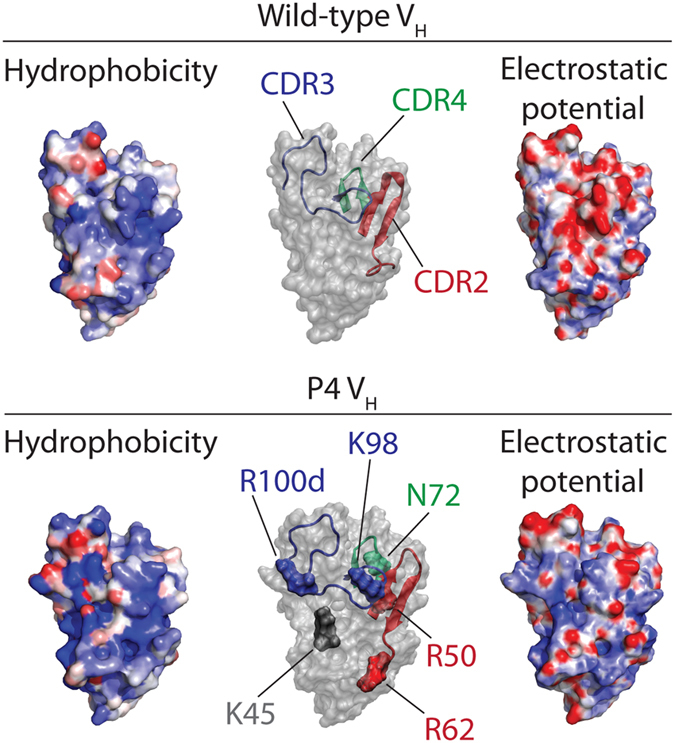
Molecular modeling of the structures and spatial variations of hydrophobicity and electrostatic potential for the wild-type and P4 V_H_ domains. Hydrophobicity maps of wild-type (top left) and P4 (bottom left) V_H_ domains were generated by modeling their interactions with methane-like probes. Atoms are colored according to the frequency of interaction with hydrophobic probes within a cutoff distance of 0.5 nm, as observed in the molecular dynamics simulations. Hydrophobic regions that favor interactions with methane-like probes are highlighted in red and hydrophilic regions that disfavor such interactions are highlighted in blue. The dominant structures of the wild-type (top center) and P4 (bottom center) V_H_ domains are shown in transparent grey, which were obtained using molecular dynamics simulations. The CDR2 (red), CDR3 (blue) and CDR4 (green) loops are highlighted. The key P4 affinity mutations (bottom center) are colored and labeled. Electrostatic potentials for wild-type (top right) and P4 (bottom right) domains were obtained using an adaptive Poisson-Boltzmann solver. The electrostatic potential maps are scaled from −10 to +10 k_b_T/e_c_, the negative electrostatic potential is highlighted in red, and the positive potential is highlighted in blue.

We next performed hydrophobic mapping of the V_H_ surfaces by computing their propensity to interact with small (spherical) hydrophobic probe particles. This approach characterizes context-dependent hydrophobicity of protein surfaces by monitoring the local density of hydrophobic particles near them^23^. These calculations revealed a hydrophobic pocket in the P4 CDR3 near the R100d mutation (Figs 7 and S5). Interestingly, R100d is the most important affinity mutation (Fig. 5). In addition, we used an adaptive Poisson-Boltzmann solver to calculate the electrostatic potential of the V_H_ surfaces (Fig. 7). Due to several of the positively charged mutations (K45, R50, R62, K98 and R100d) as well as a mutation that neutralized negative charge (N72), the surface of P4 displayed increased positive electrostatic potential at the former V_H_/V_L_ interface. The increase in positive charge for P4, especially near CDR3, appears to contribute to improved V_H_ binding to the negatively charged Aβ peptide.

### Affinity/stability trade-offs for evolved P4 variable domain

We next sought to determine if mutations acquired during the early rounds of affinity maturation contribute to maintaining thermodynamic stability of the matured P4 V_H_ domain. To test this hypothesis, we first expressed the additional nine P4 reversion mutants as autonomous V_H_ domains in bacteria. Most of the V_H_ domains expressed well (purification yields of 10–30 mg/L relative to 10 mg/L for P4) with the exception of the I100h mutant (purification yield of 2 mg/L). We speculate that the low expression level of the I100h variant is due to three consecutive isoleucine residues in CDR3 (100f-III-100h) that form an extremely hydrophobic patch (100e-GIIIA-100i). Nevertheless, all of the purified V_H_ domains ran as single bands on SDS-PAGE gels (Fig. S6).

**Figure 8:**
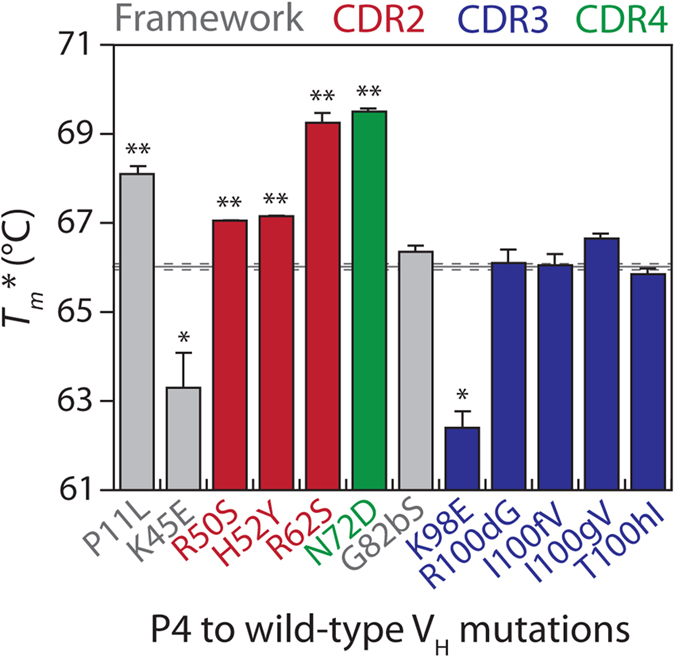
Thermodynamic stability analysis of P4 V_H_ variants with single wild-type reversion mutations. The stability of P4 variants containing single wild-type reversion mutations were evaluated as described in Fig. 3. The reversion mutations are highlighted in grey (framework residue), red (CDR2), blue (CDR3) and green (CDR4). The measurements for the P4 mutants are averages of two repeats and the error bars are standard deviations. The solid and dotted lines represent the average and standard deviation of the P4 V_H_ measurements (n = 2). A two-tailed Student’s *t*-test was used to judge statistical significance [*p*-values < 0.05 (*) or 0.01 (**)].

To evaluate the impact of each of the reversion mutations on the stability of the P4 V_H_ domain, we measured the thermal stabilities of the mutants using circular dichroism (Figs 8 and S7). We generally found that the effect of each mutation on stability in the context of the P1, P2 or P3 V_H_ domains (Fig. 3) was similar to its effect in the context of P4 (Fig. 8). For example, the destabilizing mutation P11 in the context of P1 was also destabilizing in the context of P4 (*p*-value of 0.01), while the stabilizing mutation K45 in P1 was also stabilizing in P4 (*p*-value of 0.04). Likewise, the destabilizing mutations R62 and N72 in P2 were also destabilizing in P4 (*p*-values < 0.003), and the destabilizing mutation R50 in P3 was also destabilizing in P4 (*p*-value of 0.006). One exception was that the mutation H52 was not destabilizing in P2 (*p*-value of 0.49) but was destabilizing in P4 (*p*-value of 0.005). We also found that the stabilizing mutations (K45 and K98) were primarily dependent on positive charge and could be replaced by single arginine mutations without impacting stability (*p*-values > 0.3). Moreover, the stabilizing K45 and K98 mutations in P4 were also stabilizing in the wild-type V_H_ domain used to generate the P-series variants (*p*-values < 0.04), while the most destabilizing mutations (P11, R62 and N72) were also destabilizing in the wild-type domain (*p*-values < 0.02; Figs S8, S9 and S10).

**Figure 9:**
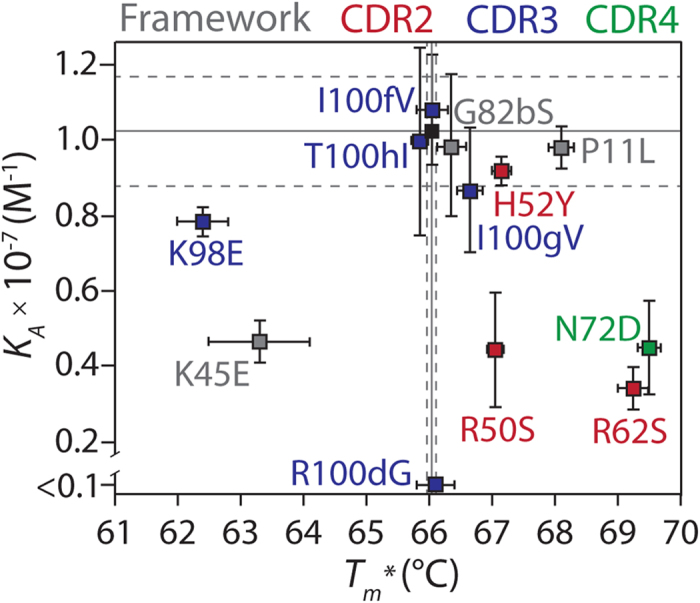
Comparison of the impact of single wild-type reversion mutations on the binding affinity and thermodynamic stability of the P4 V_H_ domain. The affinity (*K*_*A*_) and stability (*T*_m_^*^) measurements for the P4 variants containing single wild-type reversion mutations were evaluated as described in Figs 2 and 3, respectively. Decreases in V_H_ affinity or stability due to reversion mutations signify that the P4 mutations contribute positively to affinity or stability in the corresponding V_H_ domain (and vice versa for increases in V_H_ affinity or stability due to reversion mutations). The reversion mutations are highlighted in grey (framework residue), red (CDR2), blue (CDR3) and green (CDR4). The *K*_*A*_ measurements for the P4 mutants are averages of four to five repeats, while the *T*_m_^*^ measurements are averages of two repeats (the error bars are standard deviations). The solid and dotted lines are the averages and standard deviations (respectively) for the original P4 V_H_ domain (seven repeats for the association constant, two repeats for the apparent melting temperature).

We next evaluated potential affinity/stability trade-offs for the P4 reversion mutations (Fig. 9). Notably, half of the mutations that contributed most to affinity (i.e., affinity was reduced upon reversion to wild-type) were destabilizing (i.e., stability was increased upon reversion to wild-type). These destabilizing mutations included mutations in CDR2 (R50 and R62) and CDR4 (N72). Interestingly, both of the stabilizing mutations (K45 and K98) positively impacted binding affinity. Moreover, the most important P4 affinity mutation (R100d in CDR3) did not alter stability. More generally, we found that transferring the six affinity mutations (K45, R50, R62, N72, K98 and R100d) onto the wild-type V_H_ scaffold – including the two mutations that also increased V_H_ stability (K45 and K98) – resulted in an apparent stability that was significantly higher than P4 (apparent *T*_*m*_ of 70.5 ± 0.4 relative to 66.0 ± 0.1 for P4; *p*-value of 0.005). Therefore, the addition of the other six P4 mutations (P11, H52, G82b, I100f, I100g and T100h) that collectively increase affinity by more than fourfold is destabilizing. In summary, these findings demonstrate that affinity-enhancing mutations can be destabilizing and that compensatory mutations are required to maintain thermodynamic stability during affinity maturation of antibody variable domains.

## Discussion

Our findings demonstrate that some of the key affinity-enhancing mutations accumulated during directed evolution of a human V_H_ domain are strongly destabilizing. The commonality of our findings with those for natural antibodies specific for antigens of unrelated sequence and structure (93F3, OKT3 and 48G7^15^,^16^) suggests that reshaping the antigen-binding site for high-affinity binding is generally a destabilizing process. There are several mechanisms by which affinity mutations in the CDRs may lead to reductions in antibody stability, including CDR structural changes that strain the framework, loss of stabilizing interactions (e.g., hydrogen bonds) within individual CDRs or between different CDRs and/or introduction of destabilizing interactions (e.g., steric clashes) between CDRs. Indeed, these and related mechanisms appear to explain some of the destabilizing effects of affinity mutations in natural antibodies^15^,^16^.

Notably, most of the mutations in P4 are located in or near loops that connect β-strands regardless of their role in affinity or stability. Natural antibodies have also been found to accumulate both affinity and stability mutations within their loops (especially the CDRs), suggesting that these loops can mediate both properties in constructive and detrimental ways^15^,^16^. Directed evolution and protein engineering studies aimed at increasing antibody stability frequently identify stabilizing mutations within or near antibody loops^24^,^25^,^26^,^27^,^28^,^29^, similar to the stabilizing mutation we identified in CDR3 (E98K). It is also interesting that we and others have identified stabilizing lysine mutations (E45K for P4, E42K for the 48G7 antibody^16^) near the base of the β-strand leading to CDR2, which may suggest a generic type of stabilization due to the removal of negative charge and/or introduction of positive charge in this region.

It is also interesting that P4 accumulated both positively charged and hydrophobic mutations in a manner that is generally similar to human antibodies that have undergone somatic hypermutation *in vivo*^30^,^31^. The six most important affinity mutations acquired by P4 (K45, R50, R62, N72, K98 and R100d) involved a shift toward more positive charge at the former V_H_/V_L_ interface and the formation of a hydrophobic pocket in CDR3 (Figs 7 and S5). Interestingly, sequences of diverse human antibodies that have undergone somatic hypermutation show shifts toward increased hydrophobic solvent-accessible surface area and more positive charge (or less negative charge) in the CDRs relative to human germline antibodies^30^. Further, deep sequencing analysis of circulating human antibodies *in vivo* reveals that heavy chain CDR3 has more positive charge than expected based on theoretical predictions^31^. The similarity of our findings with those for natural human antibodies suggests that our findings may be due (at least in part) to factors that are more general than those due to the specific antigen recognized by the P4 V_H_ domain (Aβ is hydrophobic and negatively charged at neutral pH).

Our observation that affinity-enhancing mutations in HCDR3 have little impact on stability also deserves further consideration. The fact that HCDR3 tolerates a large amount of diversity in terms of both loop length and sequence suggests that V_H_ folding and stability are weakly dependent on the specific sequence and structure of this loop^32^. Indeed, this has been elegantly demonstrated for the parental V_H_3 domain (B1a) used in this work^18^. Shotgun alanine-scanning analysis revealed that most positions in HCDR3 displayed little preference for alanine or the wild-type residue. This suggests that the stability of the B1a domain is weakly impacted by HCDR3 sequence, which is consistent with our findings. These findings are also consistent with previous work demonstrating that HCDR3 of antibodies and antibody fragments can be grafted with peptides and small proteins without significant reductions in stability^33^,^34^,^35^,^36^,^37^,^38^,^39^,^40^,^41^.

Our findings that certain CDRs are more susceptible to accumulating destabilizing affinity mutations (HCDR2 and HCDR4) also share similarities with findings for some natural antibodies^16^. For example, of the ten somatic mutations accumulated by the hapten-specific antibody 48G7, the three most important affinity mutations are located in HCDR2, HCDR4 and LCDR2. Notably, these affinity mutations strongly destabilized the germline antibody (apparent melting temperature was reduced by ~18 °C for a germline variant containing the three key affinity mutations). This is generally similar to our observations for the HCDR2 and HCDR4 affinity mutations, as R62 (HCDR2) and N72 (HCDR4) reduced the wild-type V_H_ stability by ~4 and ~7 °C, respectively (Fig. S10). These findings are also consistent with previous analysis of V_H_3 libraries before and after enrichment for binding to Protein A (which recognizes stably folded V_H_3 domains), as HCDR2 and HCDR4 are less able to accommodate sequence diversity while maintaining stability relative to HCDR3^32^.

Our directed evolution approach used for isolating the P4 V_H_ domain by co-selecting affinity and stability mutations^17^ shares similarities with previous studies aimed at using natural antibody diversity and various display methods to optimize antibody affinity and/or stability^26^,^42^,^43^,^44^,^45^,^46^,^47^,^48^,^49^. For example, our approach of grafting peptides into CDR3 and selecting for sets of mutations that contribute to affinity and stability using yeast surface display shares some commonalities with approaches in which CDRs are grafted onto stability-engineered antibody scaffolds and mutations are selected that optimize affinity and stability using display methods^26^,^42^,^43^. Moreover, others have demonstrated the use of natural sequence diversity in the framework regions to identify mutations that primarily contribute to enhanced stability^44^, as we observed for the framework mutation K45 and others have observed for somatic framework mutations in natural antibodies^16^. These and other efforts aimed at creating antibody libraries based on natural patterns of framework and CDR diversity^45^,^46^,^47^,^48^ may help overcome the susceptibility of *in vitro* selection strategies to yield antibodies with poor biophysical characteristics^50^,^51^,^52^.

In a broader sense, trade-offs between protein function and stability extend beyond antibodies. Directed evolution efforts aimed at improving several non-antibody proteins for either increased stability or function have resulted in evolved variants with increased function but reduced stability (or vice versa)^53^,^54^,^55^,^56^. Moreover, mutations within the active sites of enzymes are often acquired at the expense of stability^5^,^6^,^7^,^8^. Conversely, many mutations that stabilize enzymes reduce their activity^4^,^57^,^58^. While antibodies have been suggested to possess optimal folds for protein engineering^59^ and have been assumed to be less susceptible than enzymes to activity/stability trade-offs, our findings demonstrate that functional (affinity) mutations can be strongly destabilizing and antibodies (like enzymes) require compensatory mutations to maintain thermodynamic stability.

## Conclusions

We find that mutations acquired during extensive mutagenesis and affinity maturation of antibody variable domains can be strongly destabilizing and acquisition of compensatory mutations is important for maintaining thermodynamic stability. These findings are consistent with those for natural antibodies^15^,^16^ and suggest that the process of forming the antigen-binding site during affinity maturation is generally destabilizing. Moreover, our observation that affinity mutations in HCDR3 weakly impact stability suggests that synthetic antibody libraries with diversity primarily in HCDR3 may be useful for minimizing affinity/stability trade-offs^18^,^32^,^60^,^61^. Our findings also suggest that antibody libraries with significant CDR diversity outside HCDR3 may require compensatory mutations to maintain thermodynamic stability. Future efforts should focus on refining and using directed evolution methods that mimic the natural process of somatic hypermutation and which enable efficient selection of both affinity and stability mutations during antibody discovery and affinity maturation.

## Materials and Methods

### Cloning of antibody variants

The grafted Aβ33–42 wild-type (WT) and P-series V_H_ domains (P1, P2, P3 and P4) were cloned into the pCTCON2 yeast display and pET-17b bacterial expression vectors as described previously^17^. Individual point mutations were generated via site-directed mutagenesis using *PfuUltra* II Fusion Polymerase (600850, Agilent Technologies) and custom DNA primers encoding the appropriate nucleic acid mutation (Integrated DNA Technologies). Wild-type and mutant Aβ33–42 V_H_ domains were generated via PCR assembly of overlapping DNA primers^62^. Afterward, the V_H_ genes were either digested (*NheI* and *SalI*) and ligated into the pET-17b bacterial expression vector or ligated (without digestion) into the pCTCON2 yeast display vector via homologous recombination. The Aβ33–42 WT and P4 V_H_ domains were also cloned into a modified form of the pCHA-FcSup-TAG yeast expression vector for soluble secretion of the autonomous V_H_ domains from yeast^63^. Both of the V_H_ genes were assembled from oligonucleotide primers and cloned into the yeast expression vector via homologous recombination.

### Yeast surface display

The V_H_ domains were displayed on the surface of *S. cerevisiae* (EBY100) by genetically fusing them to the C-terminus of Aga2^20^. Yeast cells transformed with pCTCON2 plasmids encoding Aga2-V_H_ fusions were grown overnight in 5 mL of low pH SD-CAA medium (20 g/L of dextrose, 6.7 g/L of yeast nitrogen base without amino acids, 5 g/L of casamino acids, 14.7 g/L of sodium citrate and 4.3 g/L of citric acid) at 30 °C and 220 rpm to an OD_600_ of 1–2^64^. To induce surface display of Aga2-V_H_ proteins, the cells were pelleted at 2,500 × g for 5 min and re-suspended in 5 mL of SG-CAA medium (20 g/L galactose, 6.7 g/L of yeast nitrogen base without amino acids, 5 g/L of casamino acids, 8.56 g/L of NaH_2_PO_4_ · H_2_O and 5.4 g/L of Na_2_HPO_4_). The SG-CAA cultures were grown for 16–18 h at 30 °C and 220 rpm to allow expression of the displayed V_H_ domains. Both SD-CAA and SG-CAA media were supplemented with 1% penicillin/streptomycin and 100 μg/mL of both ampicillin and kanamycin.

Equilibrium association constant (*K*_*A*_) values of the V_H_ domains were measured on the surface of yeast via flow cytometry. Immediately prior to such analysis, 10^7^ displaying cells were washed twice in 1 mL of PBS-B (PBS + 1 g/L BSA). A fraction of the cells (10^6^) were then incubated in 250 μL of PBS-B containing various concentrations of biotinylated Aβ and allowed to bind for 3 h at 25 °C. Afterward, the yeast cells were pelleted and subsequently washed with 0.3 mL of PBS-B buffer. Cells were then labeled with Alexa Fluor 647-conjugated streptavidin (1:100 dilution) in 200 μL of PBS-B for 5 min on ice. After secondary labeling, the yeast cells were washed in 0.3 mL of PBS-B and antigen binding was measured via the mean fluorescence in the APC channel on a BD LSRII flow cytometer. The mean APC fluorescence values were then fit to a binding model where APC = APC_min_ + APC_sat_ [Aβ]/([Aβ] + *K*_*D*_) + APC_ns_ [Aβ]. APC_min_ is the minimum APC value, APC_sat_ is the APC saturation value, *K*_*D*_ is the equilibrium dissociation constant, APC_ns_ is the APC signal for the non-specific binding of Aβ42, and [Aβ42] is the Aβ42 concentration. Model fitting was performed in Microsoft Excel using the solver tool to minimize the mean squared error between the experimental and predicted APC values. The association constants (*K*_*A*_) were determined by taking the inverse of the fitted *K*_*D*_ value.

### Bacterial expression and purification

Bacterial expression of the V_H_ domains was performed using the pET-17b expression vector. The V_H_ domains contained N-terminal PelB leader sequences to direct periplasmic expression as well as C-terminal triple FLAG tags (for detection) and a heptahistidine tag (for purification). Expression plasmids were transformed into BL21(DE3) pLysS cells (200132, Agilent Technologies) via heat shock, and selected on LB agar plates with 100 μg/mL ampicillin after incubation at 37 °C for 18 h. Afterward, the agar plates were scraped to collect transformed colonies and inoculated into 200 mL of autoinduction media^65^ supplemented with ampicillin (100 μg/mL) and chloramphenicol (35 μg/mL). Expression was carried out for 48 h in 1 L shake flasks at 30 °C and 225 rpm.

The cells were then discarded and the V_H_ domains were purified from the supernatant using 3 mL of Ni-NTA agarose resin (1.5 mL of settled resin; 30230, Qiagen). After 18 h of mild agitation at 4 °C, the resin was collected and washed with 250 mL of PBS. The purified V_H_ domains were then eluted in 3 mL of PBS at pH 3, and neutralized to pH 7.4. Protein aggregates were removed after neutralization by centrifuging the samples at 21,000 × g for 5 min and filtering them through a 0.2 μm membrane (SLGV013SL, Millipore). Protein concentrations were quantified via absorbance measurements at 280 nm, and protein purity was evaluated via SDS-PAGE (WG1203BX10, Life Technologies) after reducing and boiling the samples. Some of the V_H_ domains were refolded by buffer exchanging them into PBS with 6.6 M GuHCl (pH 7.4) using spin columns (89893, Thermo Scientific), allowing them to unfold at 4 °C for 18 h, and then refolding them by buffer exchanging them twice into PBS (pH 7.4).

### Yeast expression of soluble V_H_ domains

Yeast expression of soluble Aβ33–42 WT and P4 V_H_ domains was performed using the modified pCHA-FcSup-TAG vector. Yeast (EBY100) were transformed with pCHA constructs and selected on SD-CAA agar plates. Positive transformants were scraped from the plate and inoculated into 200 mL of SD-CAA media in a 1 L shake flask. Cells were grown for 2 d at 30 °C and 225 rpm to an OD_600_ of ~6. Afterward, V_H_ expression was induced by replacing the growth media with 200 mL of SG-CAA media, and incubating the yeast for 3 d at 30 °C and 225 rpm. The cells were then discarded and the V_H_ domains were purified from the supernatant in the same manner as for V_H_ domains expressed in bacteria. V_H_ concentrations were analyzed using the Pierce BCA protein assay kit (23225, Thermo Scientific). The purified V_H_ domains were also treated with a protein deglycosylation enzyme mixture (P6039S, New England Biolabs), PNGase F (P0704S, New England Biolabs) or *O*-Glycosidase (P0733S, New England Biolabs) to remove glycans prior to SDS-PAGE analysis.

### Circular dichroism

The secondary structure and thermal stability of V_H_ domains were evaluated via circular dichroism (CD) using a Jasco 815 spectrophotometer. Far-UV CD spectra were collected over the range of 200 to 260 nm for V_H_ domains diluted to a final concentration of 0.2 mg/mL in water (from stock solutions ranging from 0.3 to 6 mg/mL in PBS). Ellipticity measurements were collected every 0.5 nm at a scanning speed of 50 nm/min (1 nm bandwidth and 4 s response time). Mean residue ellipticity values were calculated from averages of 10 accumulations after background subtraction of the buffer spectrum.

Thermal unfolding curves were measured by monitoring the ellipticity at 235 nm for V_H_ domains diluted to a final concentration of 0.1 mg/mL in water (from stock solutions ranging from 0.3 to 6 mg/mL in PBS). V_H_ domains were heated from 25 to 95 °C at a heating rate of 1.0 °C/min. After one cycle of heating, the protein was cooled to 25 °C for 15 min, and the melting process was repeated to evaluate the reversibility of unfolding. Ellipticity values from the first melt were used to calculate the apparent melting temperature, as described elsewhere^66^. Briefly, the ellipticities of the folded (Θ_F_) and unfolded (Θ_U_) states were fit as linear functions of temperature. The fraction folded at a given temperature (Θ_T_) was then calculated as (Θ_T_ − Θ_U_)/(Θ_F_ − Θ_U_). Linear regression of the transition region was used to calculate the apparent melting temperature. Melting temperatures were averaged for two independent protein melts, and error bars represent standard deviations.

### Modeling of V_H_ structures

The computational modeling of the wild-type Aβ33–42 V_H_ domain was performed using the MOE homology modeling package (Chemical Computing Group Inc., Montreal, Canada). The Aβ33–42 CDR3 was first grafted into an existing crystal structure of the parent B1a V_H_ scaffold (PDB: 3B9V)^18^. The protonation states were made to be consistent with neutral pH, and the V_H_ structure was solvated with approximately 17,000 water molecules in a cubic 3D periodic box (512 nm^3^). Next, the V_H_ domains were simulated using the GROMACS molecular dynamics (MD) package^67^. The AMBER99SB force field^68^ and the TIP3P model^69^ were used to represent proteins and water, respectively, and electroneutrality was maintained by the addition of Cl^−^ and K^+^ ions. Parameters for cross interactions were calculated using the Lorentz-Berthelot mixing rules^70^, and bonds containing hydrogens were constrained using the LINCS algorithm^71^. The electrostatic interactions were calculated using the particle mesh Ewald algorithm^72^. The temperature (300 K) and pressure (1 atm) were maintained using the Nose-Hoover thermostat^73^ and the Parrinello-Rahman barostat^74^, respectively.

The MD simulation run for the wild-type V_H_ domain was first equilibrated for 5 ns in the canonical (NVT) ensemble with V-rescale thermostat^75^, and further equilibrated for 5 ns in the isothermalisobaric (NPT) ensemble. In order to sufficiently sample additional conformations of the wild-type domain, a production run (100 ns) was performed in the NPT ensemble. Configurations were stored every 1 ps for analysis. The root mean squared deviation (RMSD) was calculated, and clustering analysis was performed to identify the most dominant structures using a greedy-type algorithm^76^ with a cutoff of 0.2 nm. The structure of the P4 domain was then modeled by introducing the twelve P4 mutations into the dominant structure for the simulated wild-type V_H_. Finally, the P4 domain was equilibrated, further simulated (100 ns), and analyzed as described for the wild-type V_H_ domain.

To evaluate the spatial patterns of hydrophobicity on the surfaces of the V_H_ domains, simulations (150 ns) were performed for the wild-type and P4 domains in aqueous solutions containing spherical hydrophobic particles (methane-like Lennard Jonesiums, σ = 0.3855 nm; ε = 0.694 kJ/mol, 80 particles). All probe particles were initially placed at least 1 nm away from the protein surface to avoid biased binding behavior, and the total number of probes was selected to avoid self-aggregation during the simulations. The average number of probe particles in the vicinity of each heavy atom was calculated over the simulation trajectory. Moreover, the electrostatic potential of the V_H_ domains was calculated using the adaptive Poisson-Boltzmann solver^77^ in PyMOL^78^.

### Statistical analysis

The values for *K*_*A*_ and *T*_m_^*^ were obtained by averaging data sets from multiple independent measurements. Results are presented as mean ± standard deviation. Statistical analysis was performed using a two-tailed Student’s *t*-test in Microsoft Excel. Differences between population means were noted as significant with computed *p*-values < 0.05 (*) or 0.01 (**).

## Additional Information

**How to cite this article:** Julian, M. C. *et al*. Efficient affinity maturation of antibody variable domains requires co-selection of compensatory mutations to maintain thermodynamic stability. *Sci. Rep.* **7**, 45259; doi: 10.1038/srep45259 (2017).

**Publisher's note:** Springer Nature remains neutral with regard to jurisdictional claims in published maps and institutional affiliations.

## Supplementary Material

Supplementary Information
