# Supplementary material for: Efficient affinity maturation of antibody variable domains requires co-selection of compensatory mutations to maintain thermodynamic stability
Source: Sci Rep. 2017 Mar 28;7:45259. doi: 10.1038/srep45259 (PMC5368667; doi:10.1038/srep45259)
Supplement: Supplementary Information [file srep45259-s1.pdf]

Supplementary Data

Efficient affinity maturation of antibody variable domains requires co-selection of compensatory mutations to maintain thermodynamic stability

Mark C. Julian, Lijuan Li, Shekhar Garde, Rebecca Wilen, Peter M. Tessier

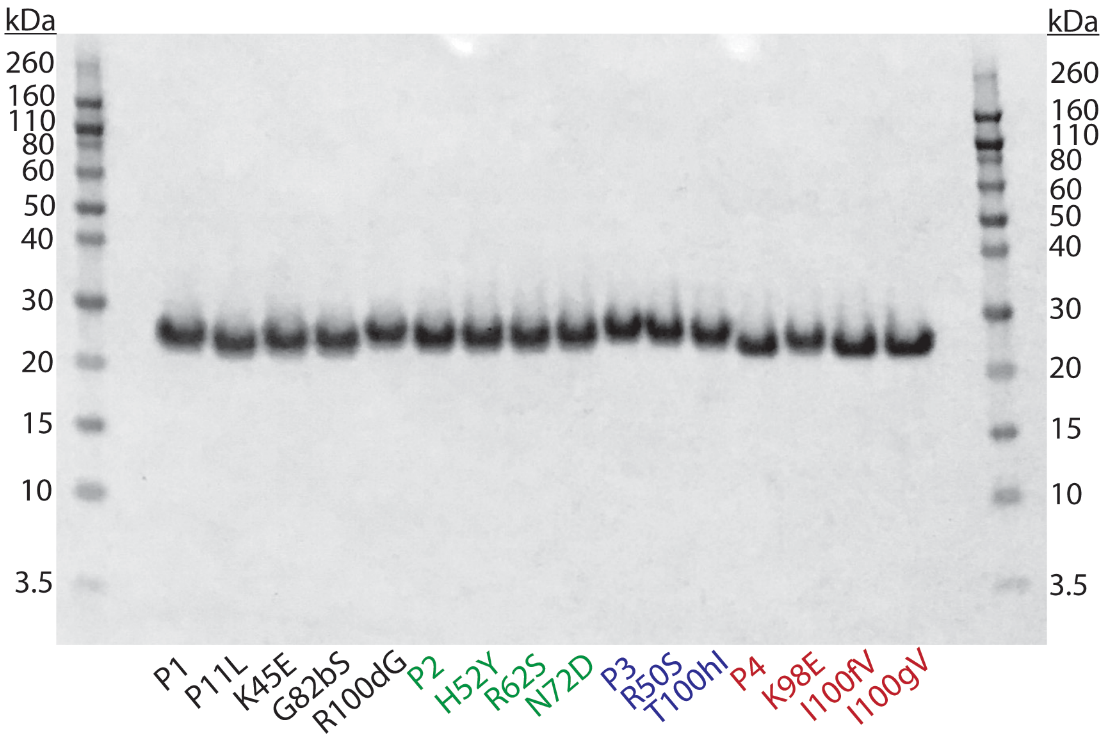

Figure S1. SDS-PAGE analysis of the P-series V_H variants with single reversion mutations. The variants were expressed as autonomous V_H domains in bacteria and purified via metal-affinity chromatography. The V_H samples were reduced (50 mM β-mercaptoethanol) and boiled prior to SDS-PAGE analysis.

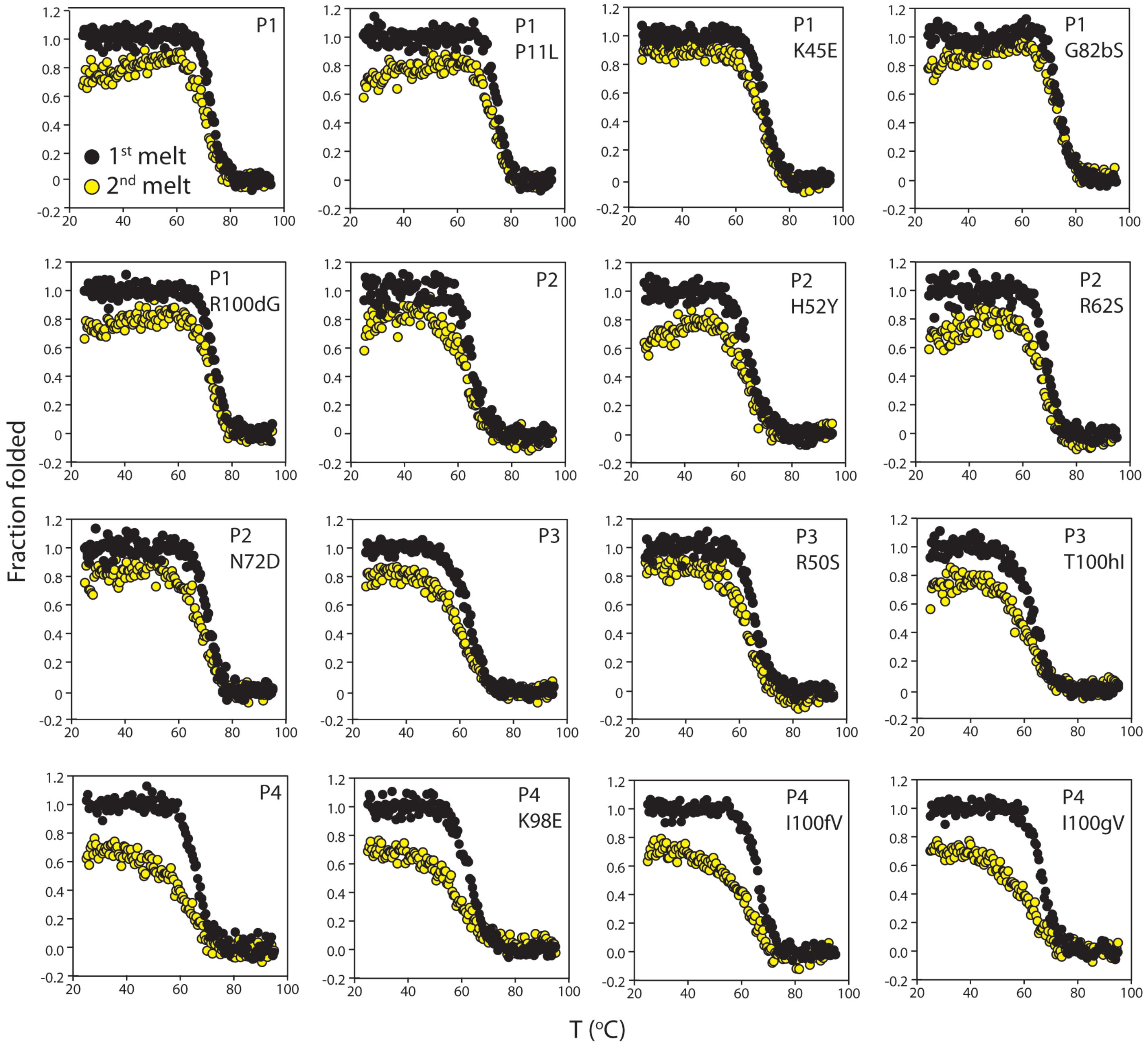

Figure S2. Evaluation of the apparent thermodynamic stability and reversibility of unfolding for the P-series V_H variants with single reversion mutations. The circular dichroism signal was monitored at 235 nm as a function of temperature and the unfolding transitions were evaluated twice for each sample to evaluate reversibility. The fraction of folded V_H domain was calculated assuming a two-state transition. The unfolding experiments were performed twice and a representative experiment is reported.

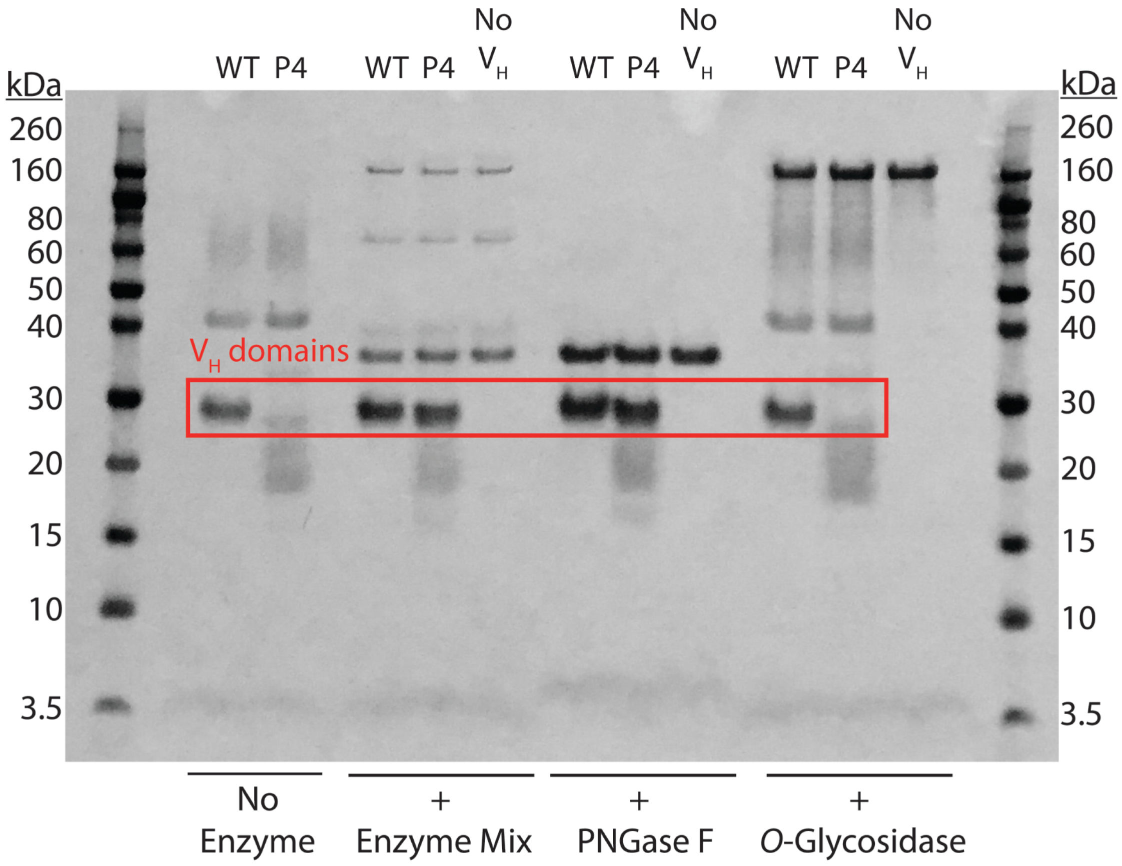

Figure S3. Glycosylation analysis of the wild-type and P4 V_H domains. The V_H domains were expressed as autonomous (non-fusion) domains in yeast. The purified V_H domains were evaluated using SDS-PAGE after incubation in the presence or absence of deglycosylation enzymes specific for N-linked glycans (PNGase F), O-linked glycans (*O*-Glycosidase) or both (enzyme mix; 37 °C, 5 h). After digestion, the samples were boiled in the presence of a reducing agent (50 mM β-mercaptoethanol). The locations of the bands corresponding to autonomous V_H domains are highlighted via a red box. The enzyme bands appear above the V_H bands.

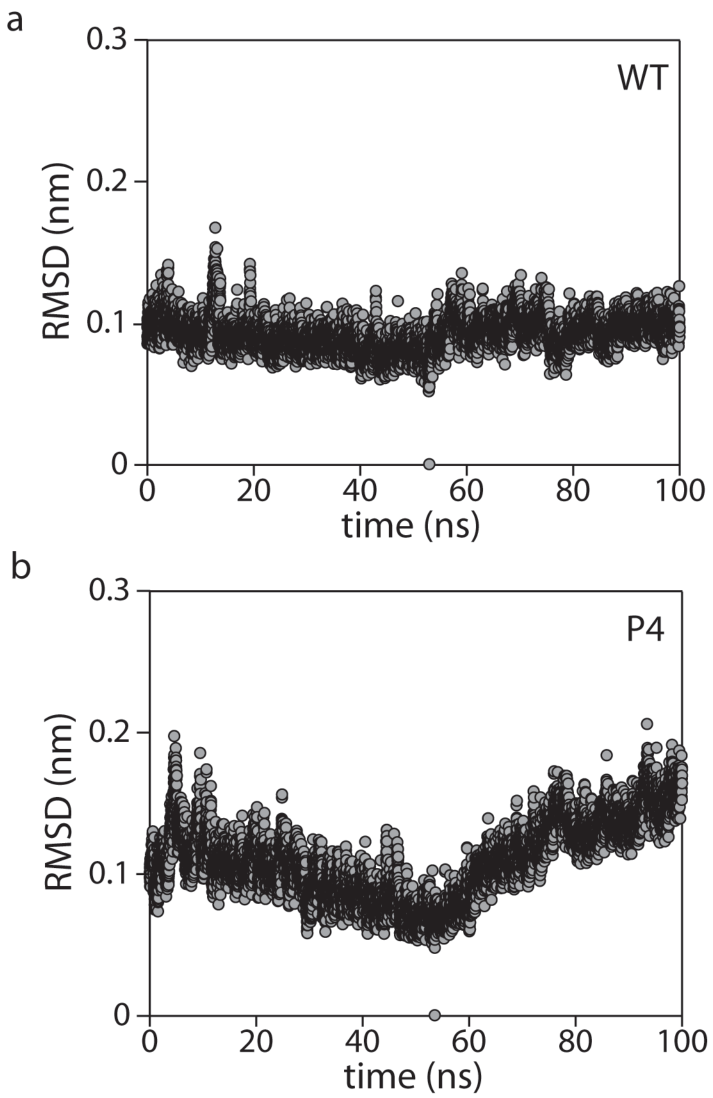

Figure S4. Evaluation of the root mean square deviations for the wild-type and P4 V_H domains during molecular dynamics simulations. The V_H domains were modeled using the GROMACS molecular dynamics package, and production runs were equilibrated for 100 ns. The root mean square deviations (RMSDs) of the backbone structures were calculated every picosecond using the dominant structure of each V_H domain.

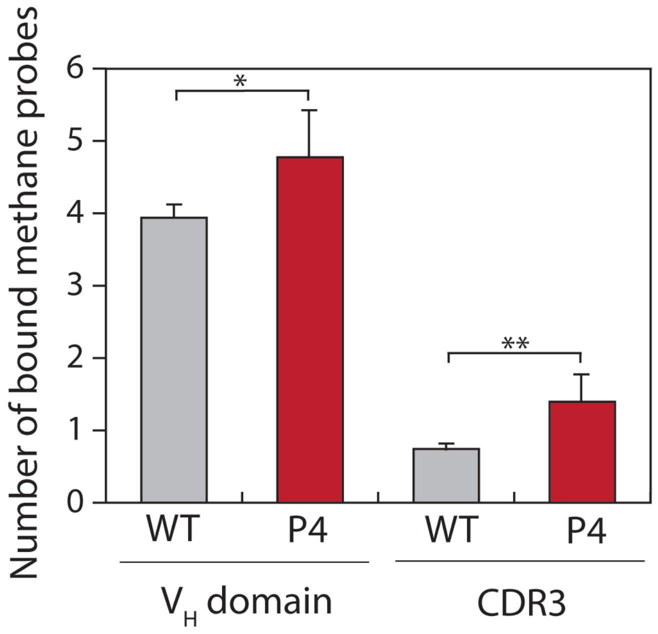

Figure S5. Computational analysis of the surface hydrophobicity of the wild-type and P4 V$_H$ domains. The V$_H$ domains were simulated in the presence of hydrophobic methane probes for 150 ns, and the average number of probes contacting the heavy atoms (within a cutoff distance of 0.5 nm) were calculated. Reported values are averages from 30 ns simulation blocks (n=5) and the error bars are standard deviations. A two-tailed Student's *t*-test was used to judge statistical significance [*p*-values < 0.05 (*) or 0.01 (**)].

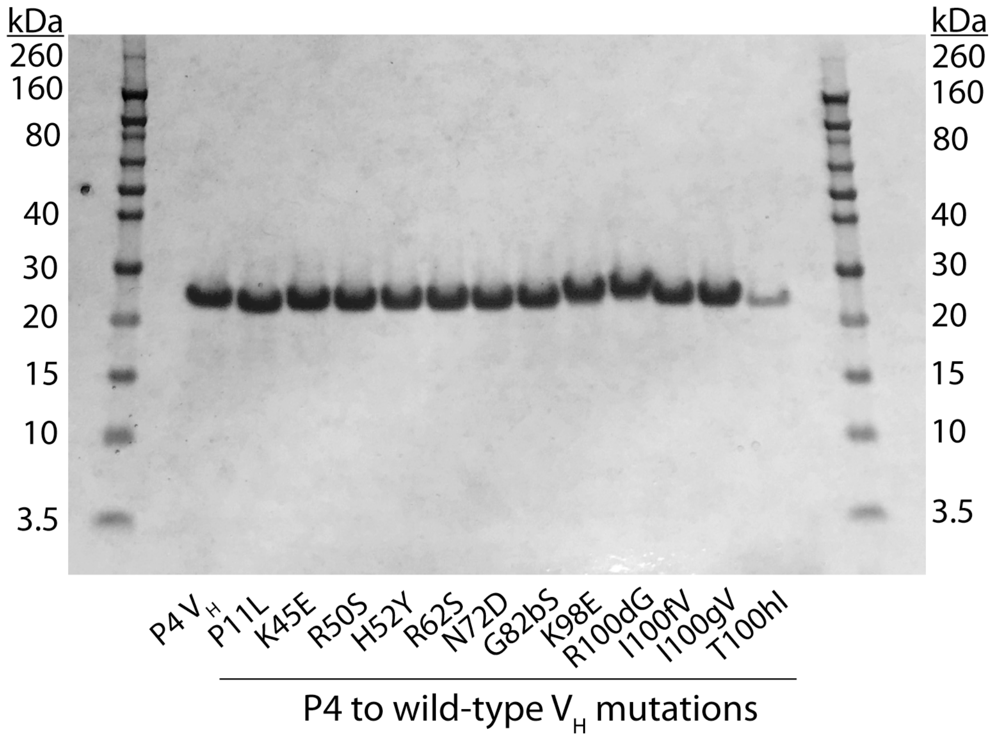

Figure S6. SDS-PAGE analysis of P4 V_H variants with wild-type reversion mutations. The P4 variants were expressed as autonomous V_H domains in bacteria and purified via metal-affinity chromatography. The V_H samples were reduced (50 mM β-mercaptoethanol) and boiled prior to SDS-PAGE analysis.

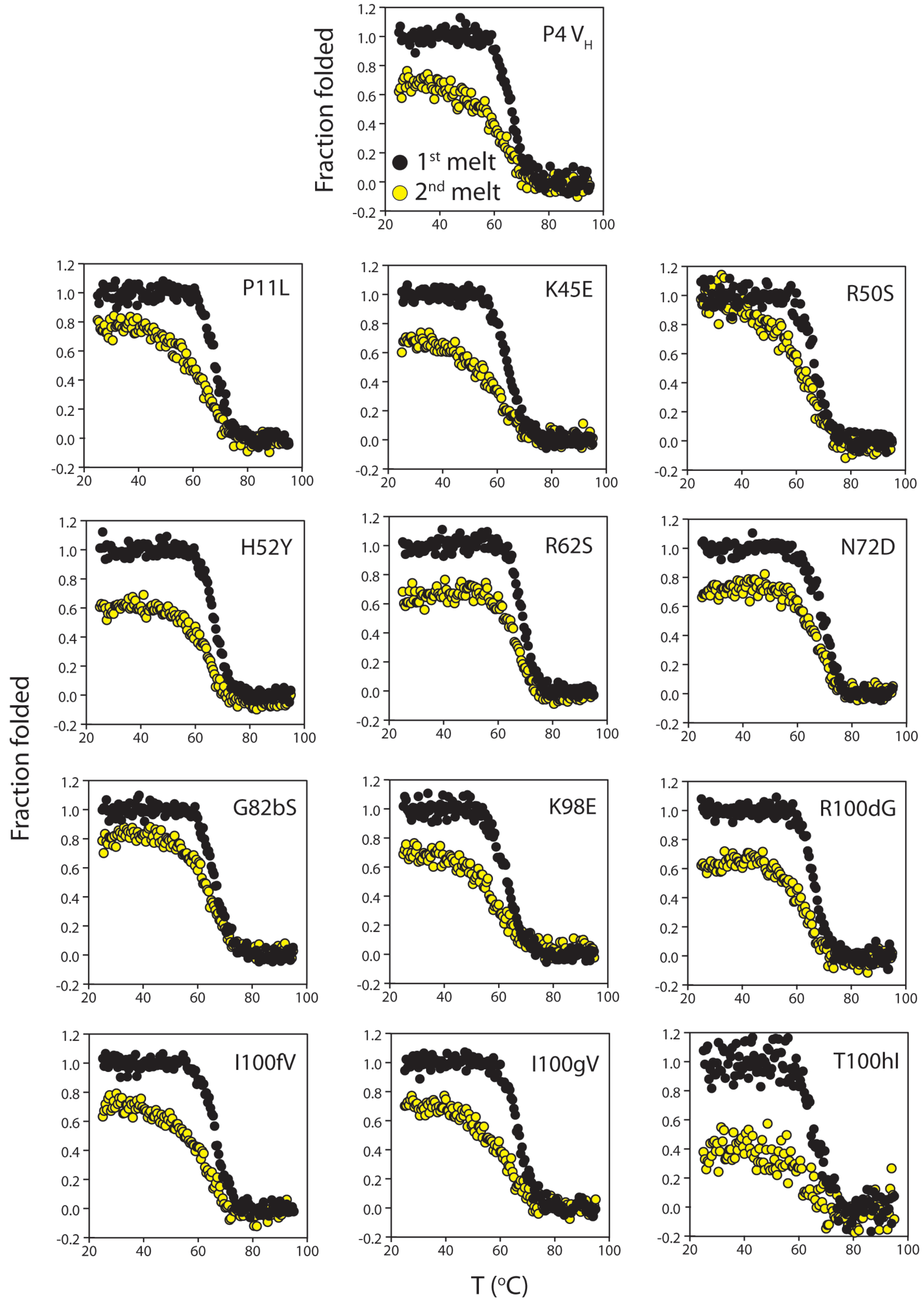

Figure S7. Evaluation of the apparent thermodynamic stability and reversibility of unfolding for the P4 V_H variants with wild-type reversion mutations. The stability and reversibility of unfolding of the purified P4 variants were evaluated as described in Fig. S2. The experiments were performed twice and a representative experiment is reported.

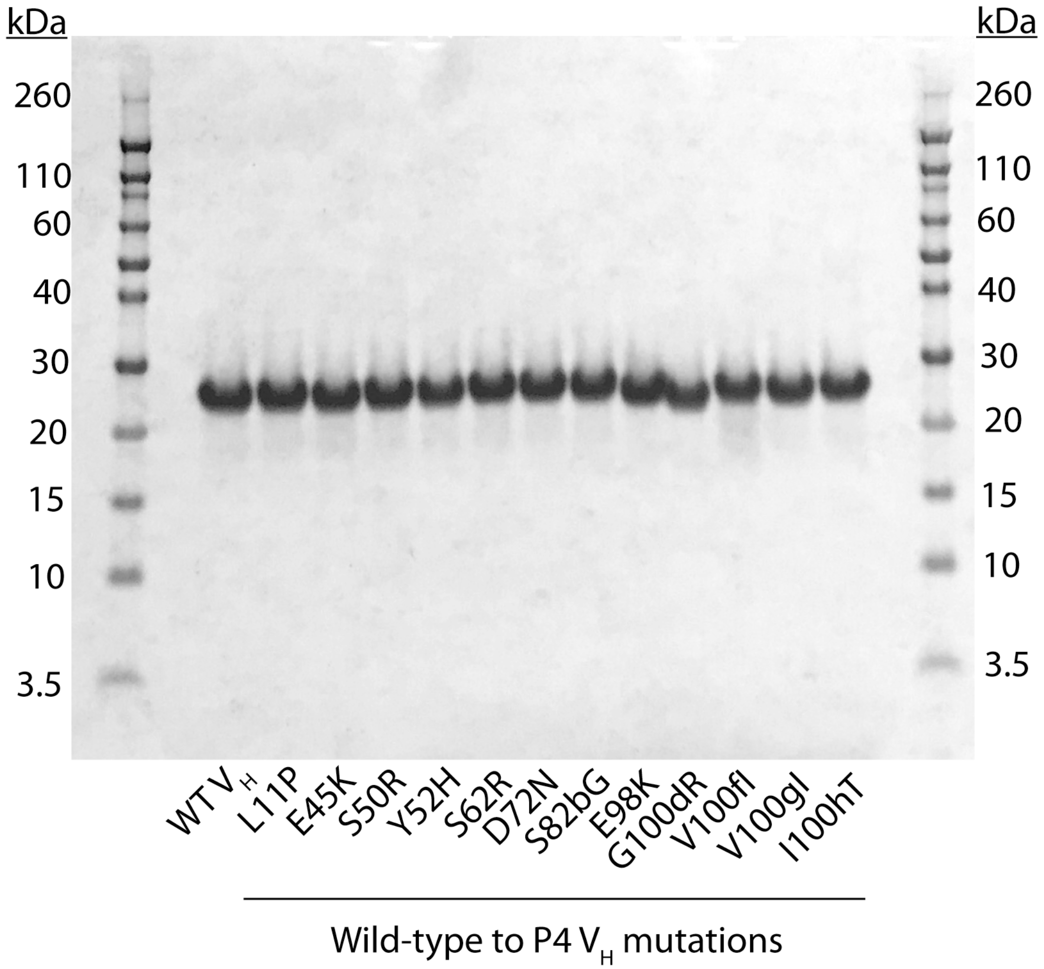

Figure S8. SDS-PAGE analysis of wild-type V_H variants with single P4 mutations. The wild-type variants were expressed as autonomous V_H domains in bacteria and purified via metal-affinity chromatography. The V_H samples were reduced (50 mM β-mercaptoethanol) and boiled prior to SDS-PAGE analysis.

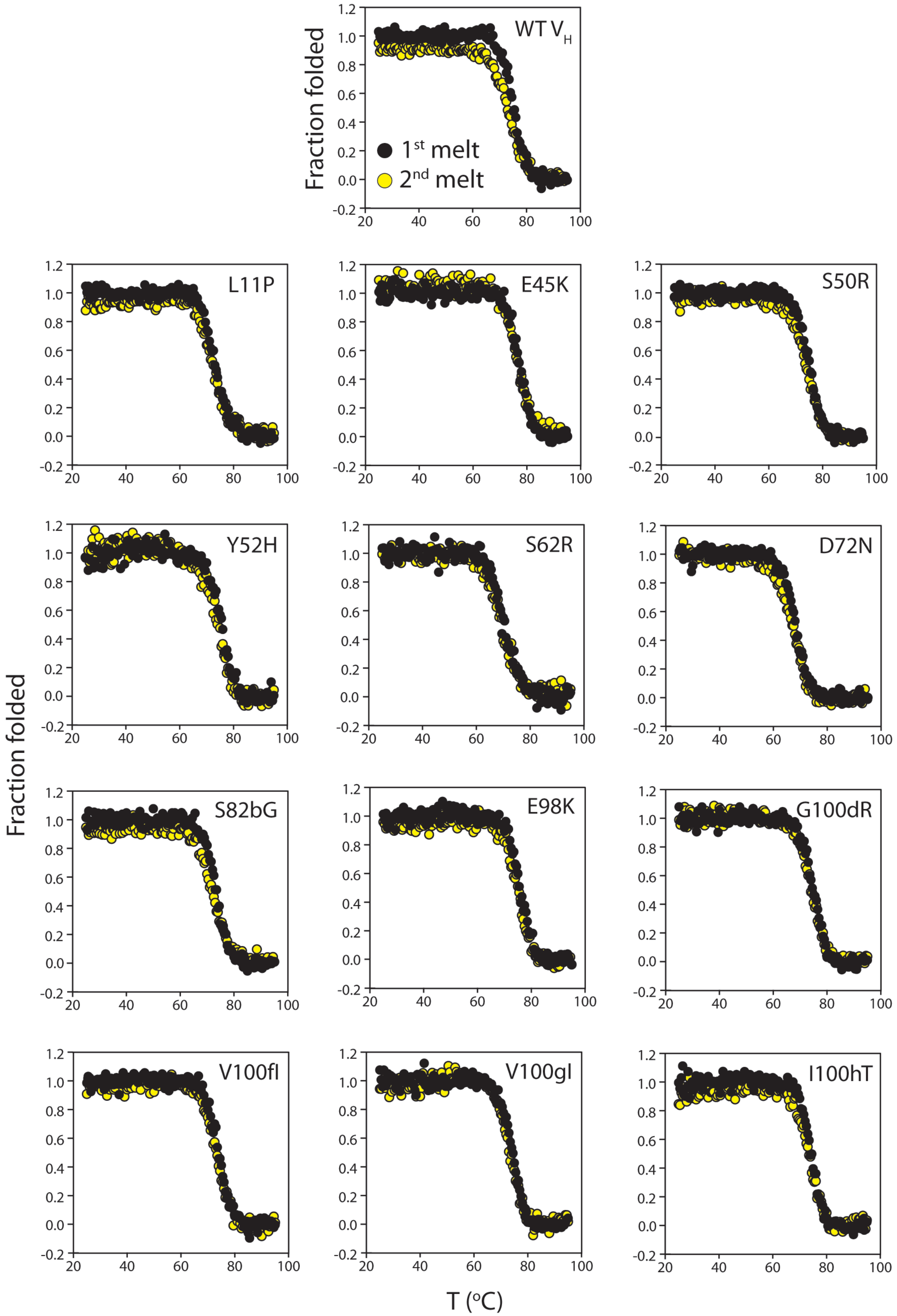

Figure S9. Evaluation of the thermodynamic stability and reversibility of unfolding for the wild-type V_H variants with single P4 mutations. The stability and reversibility of unfolding analysis was performed for the purified V_H variants as described in Fig. S2. The experiments were performed twice and a representative experiment is reported.

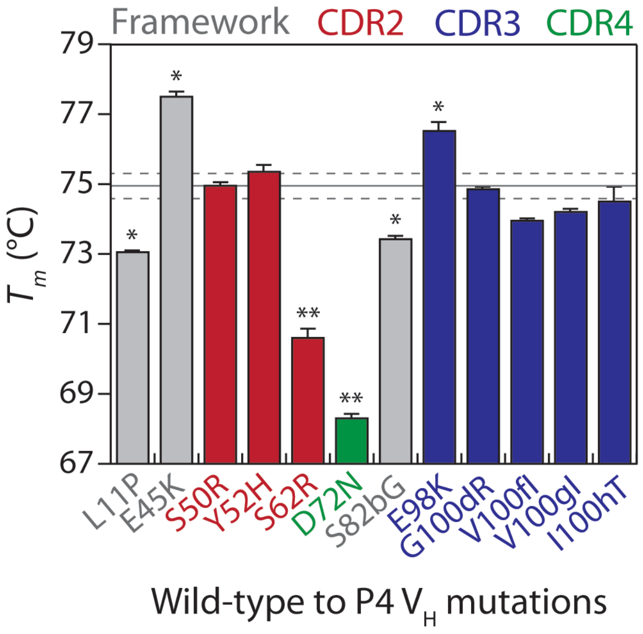

Figure S10. Thermodynamic stability analysis for variants of the wild-type V_H domain with single P4 mutations. The stability of wild-type variants containing single P4 mutations were evaluated using autonomous V_H domains produced in bacteria. Melting temperatures were calculated as described in Fig. 3. The reversion mutations are highlighted in grey (framework residue), red (CDR2), blue (CDR3) and green (CDR4). The solid and dotted lines are the average and standard deviation (respectively) for the wild-type V_H domain. The wild-type and mutant measurements were repeated twice and the error bars are standard deviations. A two-tailed Student's *t*-test was used to judge statistical significance [*p*-values < 0.05 (*) or 0.01 (**)].
